# Chronic impacts of natural infrastructure on the physical and psychological health of university students during and after COVID−19: a case study of Chengdu, China

**DOI:** 10.3389/fpubh.2024.1508539

**Published:** 2024-12-13

**Authors:** Yi Peng, Haoxing He, Bingyang Lv, Jiali Wang, Qiao Qin, Jialu Song, Yuzhou Liu, Wenjun Su, Huixing Song, Qibing Chen

**Affiliations:** ^1^Landscape Architecture College, Sichuan Agricultural University, Chengdu, China; ^2^Key Laboratory of Animal Disease and Human Health of Sichuan Province, College of Veterinary Medicine, Sichuan Agricultural University, Chengdu, China; ^3^China Construction Third Bureau First Construction Engineering Company, Wuhan, China; ^4^Sichuan Province Forestry Central Hospital, Chengdu, China

**Keywords:** natural infrastructure, COVID-19 pandemic, physical, psychological, therapeutic, well-being

## Abstract

**Introduction:**

The COVID-19 pandemic has underscored the health benefits of green spaces, yet research on how specific elements of natural infrastructure affect well-being during the pandemic has been limited.

**Methods:**

This study, conducted at Sichuan Agricultural University with 300 students in 2022, investigated how urban natural infrastructure impacts physical and psychological well-being during the pandemic. Different aspects of natural infrastructure, such as thermal comfort, air quality (negative ion concentration), and noise and light levels, varied in their positive effects on students’ health.

**Results:**

The findings revealed that 65.6% of university students felt reduced stress when engaging with outdoor spaces, and 72.8% of them renewed recognized the therapeutic value of nature.

**Discussion:**

The study emphasizes the importance of incorporating natural elements into urban planning to enhance outdoor activity and well-being, especially in post-pandemic settings. Recommendations are provided for future urban design to address the therapeutic needs of specific populations.

## 1 Introduction

The COVID-19 pandemic has had a profound impact on global physical and psychological health, drawing significant academic attention (1). Disrupting modern lifestyles, its effects on well-being have been both immediate and long-lasting (2–4). Reports indicate a sharp rise in psychological health issues among young people, with nearly half experiencing symptoms during the pandemic (5–7). The WHO (2022) reported a 25% increase in global anxiety and depression rates due to the pandemic. In China, a 2022 survey found that 6.8% of the population—around 95 million people—suffer from depression, with adolescents being particularly vulnerable. Approximately 50% of those affected are students, and 30% are under the age of 18 (8, 9).

The incidence of serious psychological health issues among university students has risen significantly in the 21st century, yet barriers often prevent them from seeking help (10). In China, about 80% of general hospitals lack dedicated psychological health departments, and insufficient mental health awareness among young people leads to low service utilization (5, 8, 11). Without timely intervention, these issues risk becoming chronic, placing economic burdens on families and society (12). Individuals aged 18–39 make up 52.7% of all psychological health cases (13).

The COVID-19 pandemic further exacerbated these issues, as physical inactivity, sedentary behavior, and disrupted sleep patterns negatively impacted cardiovascular health (14, 15). Beyond respiratory issues like pneumonia and acute respiratory distress syndrome, COVID-19 has been linked to more severe cardiovascular complications than typical respiratory infections (16–22). In China, around 330 million people suffer from cardiovascular disease (23). While psychological stress is associated with cardiovascular disease, the underlying factors remain unclear (24).

The pandemic has undoubtedly intensified these trends. The prolonged nature and global spread of COVID-19 have led many countries to implement lockdowns, including campus closures, to curb new infections (25, 26). These strict lockdowns and extended periods of indoor confinement have heightened the risk of depression and anxiety, particularly among university students, who are more susceptible to these psychological issues (27–30). Depression often co-occurs with anxiety, making university students a vulnerable group that warrants increased attention to their health and well-being (31–34).

University students are particularly vulnerable during epidemics, as disruptions to their academic and social lives, coupled with uncertainty about global health issues, can intensify existing challenges (35–37). Even before the pandemic, students often faced psychological crises due to academic stress, social pressures, future uncertainties, and unhealthy lifestyles (31, 38–40). Recent reports show a troubling increase in depression, anxiety, and suicidal thoughts among students, with global studies indicating depression prevalence rates ranging from 10 to 85%, and an average of 30.6% (39, 41).

The COVID-19 pandemic has exacerbated disruptions in interpersonal relationships, health, well-being, and academic activities, intensifying psychological health issues like major depressive disorder (MDD) and generalized anxiety disorder (GAD) among university students (42, 43). As it disrupted students’ daily lives, widespread home quarantine and social distancing measures became common (44, 45). Many educational institutions implemented partial or complete lockdowns, shifting from in-person to virtual learning formats (46, 47). Although some research has highlighted the importance of psychological health in pedagogical relationships, most studies have focused on offline teaching contexts (48–54).

Increased time spent at home (55, 56) and heightened screen time (57) have led to reduced physical activity (58–64), raising the risk of cardiovascular issues such as obesity, hypertension, and insulin resistance (65–67). These conditions and indirect factors can contribute to psychiatric problems among university students (36). Cross-national studies have indicated an increased risk of Psychiatric disorders, including post-traumatic stress disorder (PTSD) (68), anxiety (69), and complex post-traumatic stress disorder (CPTSD) (70), as well as a heightened risk of suicide following the pandemic (71–74). During the pandemic, 21.3% of university students reported mild anxiety, 2.7% moderate anxiety, and 0.9% severe anxiety (35).

Amid the escalating health impacts of the pandemic, this study explores how university students can enhance their physical and mental well-being through interactions with urban natural infrastructure. Research worldwide has highlighted the critical role of access to urban green spaces (UGS) in mitigating the challenges posed by the pandemic (75). Studies conducted in diverse locations, including the United Kingdom (75), Tokyo (76), Oslo (77), Italy (78), and Mexico City (79), consistently demonstrate that utilizing urban green spaces helps alleviate pandemic-related stress.

Further evidence underscores the positive impact of direct contact with urban green spaces on mental health (80, 81). This study examines how natural infrastructure, as an ecosystem service within urban green spaces, influences human well-being both directly and indirectly. These effects encompass short-term enhancements in physiological resilience and long-term psychological health benefits (81, 82). This focus is particularly relevant during the pandemic, when university students may have relied more heavily on nearby natural infrastructure to mitigate the adverse psychological effects of isolation and social restrictions. Moreover, in the post-pandemic period, lingering disparities in recovery highlight the importance of addressing residual mental health challenges through these therapeutic benefits.

Natural infrastructure encompasses diverse biophysical structures and ecological processes that constitute a city’s “green infrastructure” (78). Against the backdrop of increasing global health awareness, the concept of natural infrastructure has evolved beyond its traditional role as therapeutic spaces. It now emphasizes the creation of empowering landscapes that promote physical health and meet daily needs (82–87). Furthermore, natural infrastructure enhances the quality of life for urban residents, addressing societal demands while fostering overall urban well-being (88–93). By integrating with urban environments, it contributes to resolving physical and mental health challenges (94–97).

Scholars have increasingly focused on the therapeutic benefits of green spaces and their connection to healthcare and natural environments (98, 99). Interaction with green spaces has been shown to elicit positive physiological effects, such as reducing blood pressure, heart rate, and muscle tension (100), while also alleviating disease-related symptoms (101–103). During the COVID-19 pandemic, Muntner et al. (104) observed heightened levels of depression, stress, and loneliness among students but noted that “interacting with nature alleviated some of these negative emotions” (105–107). Similarly, Dzhambov (105) found that “green and blue spaces support psychological restoration in urban settings,” which is particularly relevant for university students aged 18–35, a group characterized by elevated anxiety and stress levels (108).

While the scientific community broadly agrees that interacting with nature can significantly improve mental health and well-being (109–111), research remains limited regarding the specific impacts of different types of natural infrastructure on university students’ physical and mental health, particularly during the COVID-19 pandemic. This gap prompts a critical question: How does natural infrastructure influence the physical and mental health of university students amidst the ongoing health crisis posed by the pandemic?

While most existing studies emphasize green coverage (112, 113), the use of green spaces (114–119), and green infrastructure (120) in enhancing mental health, comprehensive investigations into their overall impact on both physical and mental health remain relatively scarce. Moreover, there is a notable lack of research comparing the effects of different types of natural infrastructure and exploring their potential interactions.

Prior to the pandemic, most studies in this field focused on aspects such as green space perception (121), the Green Revolution (122), green metrics (123), types of green spaces (13), and the quantity of green spaces (124). These investigations gradually evolved from examining the effects of individual green spaces to exploring the multifaceted dimensions of natural environments and their impacts on physical and mental health. With the onset of the global pandemic, research priorities shifted toward assessing the role of natural environments during this unique period. However, this emerging area of inquiry remains underexplored, warranting further investigation.

This study aims to enrich existing research by exploring how Chinese university students utilized interactions with different types of natural infrastructure to address the physical and mental health challenges posed by the pandemic. To date, limited research has examined the use of urban natural infrastructure by Chinese university students during the COVID-19 period. To fill this gap, this exploratory study adopted an investigative approach to examine how various combinations of natural infrastructure influenced students’ health and well-being. Conducted at the end of 2022, the study recorded physiological and psychological indicators associated with the pandemic and examined students’ experiences within different types of natural infrastructure. We assessed how these interactions impacted their overall physical and mental health as well as their well-being.

**Figure 1:**
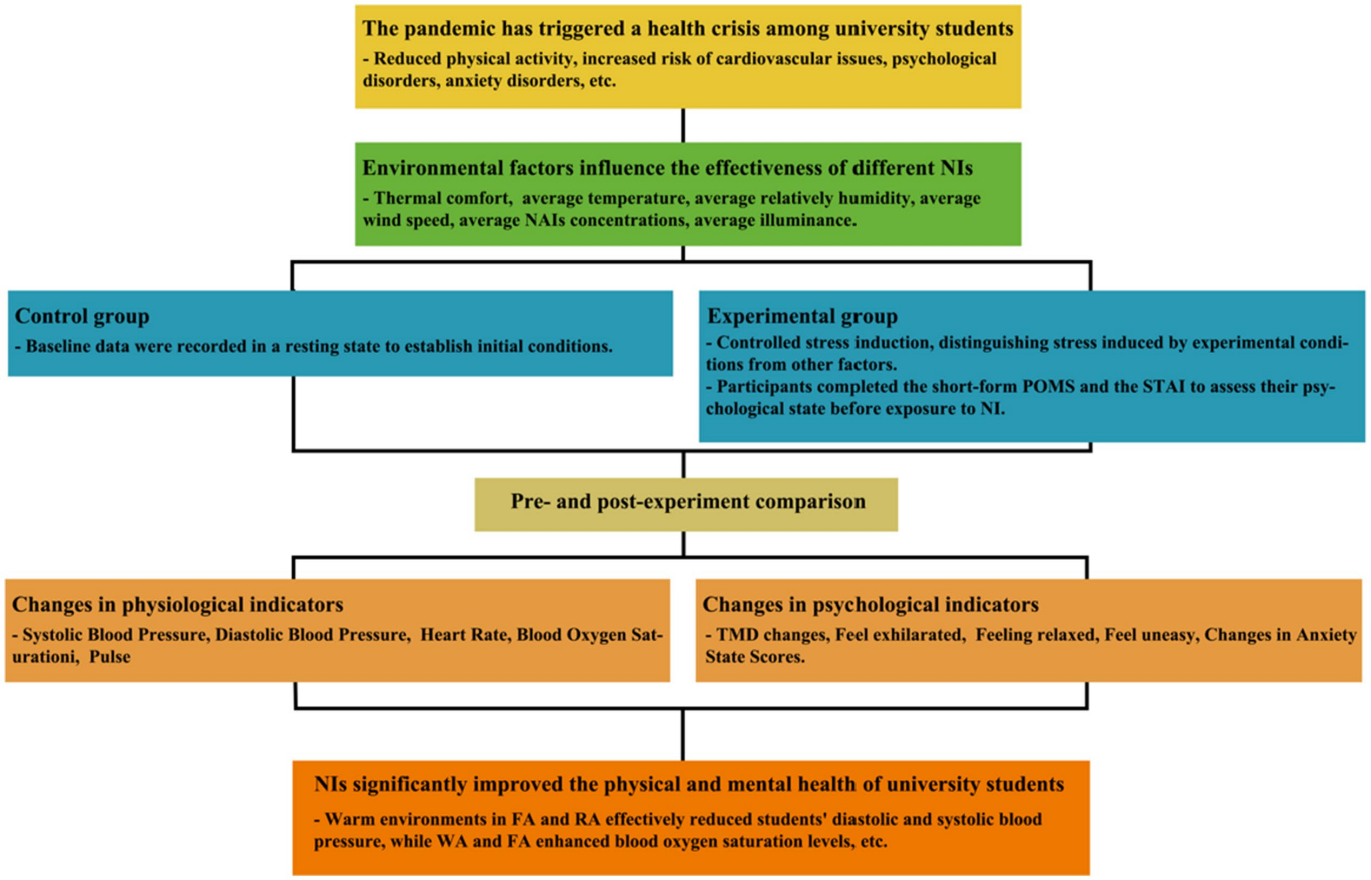
Potential mechanisms of NI in the therapeutic effects on the physical and mental health of university students.

This study will address the following three key questions based on the framework diagram (Figure 1):

1. How does participation in NI improve the physical and psychological health of Chinese university students during an epidemic?
2. Which types of NI most effectively enhance the physical and psychological well-being of Chinese university students during an epidemic?
3. To what extent does participation in different NIs contribute to the recovery of physical and psychological health among Chinese university students during an epidemic?

## 2 Materials and methods

### 2.1 Site description

**Figure 2:**
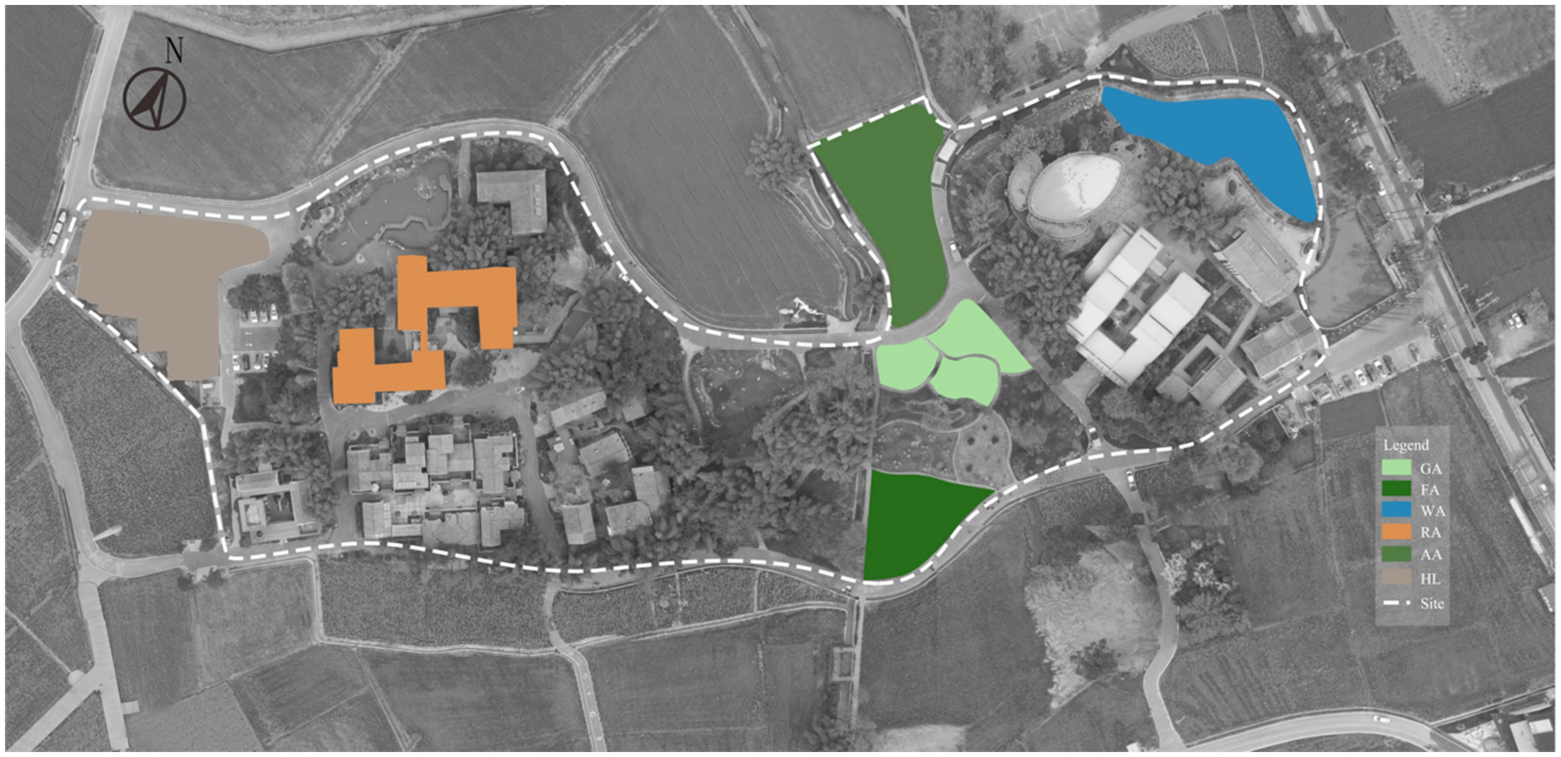
The distribution patterns of different types of NI.

The study was conducted in Pidu County, Chengdu, Sichuan Province, Southwest China. Located in the heart of the West Sichuan Plain and near the urban planning area, Pidu County is intersected by Chengdu’s fifth ring road, providing convenient access (Figure 2). We selected six types of natural infrastructure for this study, categorized into Green Infrastructure (GI), Blue Infrastructure (BI), and Hard Infrastructure (HI).

According to existing literature, the connection between these six common types of natural infrastructure and human well-being is deeply rooted. AA contribute to enhancing quality of life and promoting the sustainability of urban landscapes (125, 126) GA prove ecosystem services and support biodiversity (127), while FA play a vital role in maintaining urban environmental health (128). Additionally, WA are crucial for regulating urban climates and mitigating environmental challenges (129).

Research indicates that when university students are confined to campus or home, proximity to these green and blue spaces can reduce the risk of depression and anxiety (28, 87, 112, 130–133).

Hard Infrastructure (HI), such as Hard Landscaping (HL), represents essential physical components in modern urban environments, including roads and bridges. This study used hard infrastructure as a control group to compare the effects of green infrastructure on the physical and psychological well-being of Chinese university students.

### 2.2 Participants

Numerous studies have highlighted the significant impact of the COVID-19 pandemic on university students, contributing to both physical and psychological health issues (25–30). With their shared age and cultural backgrounds, university students provide a relatively homogeneous group for research.

This study surveyed 300 healthy, full-time students from Sichuan Agricultural University as part of the NI Therapeutic Study, achieving an 89% response rate for the 2022 questionnaire. Participants, aged 18–25 years (M = 20.9, SD = 1.88), were fluent in Chinese and recruited between October 1–20, 2022, during favorable autumn weather in Chengdu (7–18°C). Eligibility criteria excluded individuals with chronic or psychological conditions, those using psychotropic or narcotic drugs, and anyone unwilling or unable to provide informed consent. The ethics committee approved all protocols to ensure participant protection and respect.

The sample represented 22 academic disciplines, including Landscape Architecture (*n* = 130), Environmental Design (*n* = 42), Finance (*n* = 15), Horticulture (*n* = 15), Chinese Herbology (*n* = 12), and others, ensuring disciplinary diversity. The distribution of academic backgrounds was recorded to assess potential bias, confirming no significant influence on study outcomes. Of the participants, 65% (*n* = 195) identified as female and 35% (*n* = 105) as male (Table. 1).

**Table 1:** Statistical table of demographic characteristics.

Individual-level variables	Number (*N*)	Percent	Mean	SD
Age 18–25	300	100.0	21.09	1.88
18	19	6.3		
19	51	17.0		
20	69	23.0		
21	30	10.0		
22	58	19.3		
23	32	10.7		
24	32	10.7		
25	9	3.0		
Gender				
Female	105	35.0		
Male	195	65.0		
Height			M = 175.87\F = 161.53	M = 5.86\F = 4.58
Weight			M = 67.05\F = 51.04	M = 9.67\F = 5.62
Major				
Finance	15	5.0		
Environmental design	43	14.3		
Landscape architecture	121	40.3		
Grass science	6	2.0		
Environmental studies	11	3.7		
Forestry	10	3.3		
Horticulture	15	5.0		
Agricultural science	7	2.3		
Pharmacognosy	6	2.0		
Teology	12	4.0		
Financial management	2	0.7		
Investment science	14	4.7		
Animal quarantine	4	1.3		
Animal husbandry	2	0.7		
Resource science	2	0.7		
International trade	2	0.7		
Chinese herbology	12	4.0		
Aquatic conservation	3	1.0		
Zoological medicine	3	1.0		
Material science	4	1.3		
Animal science	5	1.7		
Seed Science	1	0.3		
Therapeutic feedback				
NI				
Feeling the pressure? (Pre-experience)				
Yes	134	53.6		
No	24	9.6		
Intense	24	9.6		
Moderate	68	27.2		
Feeling relief from stress? (Post-experience)				
Yes	164	65.6		
No	86	34.4		
Think therapeutic is important? (Post-experience)				
Important	182	72.8		
Moderate	68	27.2		
HI				
Feeling the pressure? (Pre-experience)				
Yes	25	50.0		
No	7	14.0		
Intense	0	0.0		
Moderate	18	36.0		
Feeling relief from stress? (Post-experience)				
Yes	33	66.0		
No	17	34.0		
Think therapeutic is important? (Post-experience)				
Important	40	80.0		
Moderate	10	20.0		

Participation was voluntary, with informed consent obtained from all participants and university officials. Students were assured of confidentiality, and their responses were not linked to academic evaluations. This rigorous approach underscores the study’s commitment to ethical research practices and diverse representation in exploring the relationship between natural environments and student well-being.

Green infrastructure included Agricultural Areas (AA), Grass Areas (GA), and Forest Areas (FA). BI includes natural or man-made systems related to water, which promote ecological diversity and enhance the quality of life for university students. In this study, BI was represented by Water Areas (WA). We focus on the overall environmental benefits of BI and their impact on the physical and psychological health of university students.

### 2.3 Data collection

This study employed a mixed-methods approach, combining both quantitative and qualitative research methodologies. The quantitative component involved correlation analysis to investigate the therapeutic effects and relationships between different types of natural infrastructure and participants’ well-being. The qualitative phase utilized questionnaires to explore the connections between natural infrastructure and the participants’ physiological and psychological states.

#### 2.3.1 Environmental data measurement

The study employed dynamic measurement techniques to assess environmental indicators relevant to human well-being, including thermal, air, light, and sound conditions. Measurements were conducted cyclically at selected locations across different geographical areas between 8 a.m. and 8 p.m. Over a one-hour period at each site, three consecutive readings were taken for each indicator after the instrument stabilized, with measurements completed within 5–8 min per site. The process was supported by three staff members to ensure accuracy and consistency.

Thermal conditions were assessed using a Taiwan Hengxin AZ8778 black ball thermometer to measure temperature (0–50°C) and relative humidity (0.1–100% RH). Wind speed (0.8–30.0 m/s) was recorded with a Sigma AS806 anemometer, while air quality was evaluated with a KEC900A Air Negative Oxygen Ion Detector. Light levels were measured using a Sigma AR813A digital illuminance meter, and noise levels (30–130 dB) were recorded with a MASTECH Huayi MS6701 digital sound level meter.

This comprehensive approach ensured precise and reliable environmental data collection, contributing to a robust evaluation of the relationship between environmental conditions and human well-being.

#### 2.3.2 Measurement of physiological and psychological data of university students

This study explored the therapeutic effects of natural infrastructure on both physical and psychological health by evaluating changes in various indicators before and after participants’ exposure to natural environments. Baseline measurements were first taken while participants were at rest to establish initial conditions.

The primary physiological parameters measured included blood pressure (systolic and diastolic), heart rate, oxygen saturation, and pulse rate. Blood pressure and heart rate were recorded simultaneously using a Fischer arm-type electronic sphygmomanometer. Normal blood pressure ranges from 90 to 140/60 to 90 mmHg (systolic/diastolic), while the pulse rate at rest should be between 60 and 100 beats per minute. Oxygen saturation was measured with a normal range of 95–100%, and heart rate was also expected to fall between 60 and 100 beats per minute.

Psychological indicators were assessed using standardized scales. The Profile of Mood States (POMS) scale, developed by McNair, Lorr, and Droppleman in 1971, is a widely recognized tool for assessing an individual’s emotional state over a specific time period (134). This self-report instrument measures six distinct mood dimensions: tension/anxiety, anger/hostility, vigor/activity, fatigue, depression, and confusion (135). In this study, POMS was used to assess changes in Total Mood Disturbance (TMD) scores and various aspects of emotional distress.

The State–Trait Anxiety Inventory (STAI), developed by Spielberger, Gorsuch, and Lushene, is another widely used self-report tool (136). It measures two types of anxiety: state anxiety, a temporary condition triggered by specific situations, and trait anxiety, a general predisposition to respond anxiously to perceived threats.

The study aimed to identify psychological changes in university students before and after exposure to natural infrastructure. An *a priori* power analysis conducted using G Power targeted a statistical power of 0.95, an effect size of 0.5, and a significance level of 0.05. This analysis determined that at least 105 participants per group were needed, yielding a total sample size of 210. To ensure statistical reliability, a sample size calculation (Equation 1) with an expected 20% response rate, a 5% margin of error, and a 95% confidence level indicated that a minimum of 246 samples was required for reliable results.


(1) $$n=\frac{Z^{2}\times p\times \left(1-p\right)}{E^{2}}$$

### 2.4 Procedure

Participants were first briefed on the testing procedures and safety measures, followed by the collection of their basic information and preliminary questionnaires. Baseline data was recorded while participants were at rest to establish initial conditions, ensuring consistency and comparability for assessing physiological and psychological changes before and after stress induction. This baseline data was crucial for understanding participants’ normal state without external stressors.

The Trier Social Stress Test (TSST) was then administered, involving 30 verbal arithmetic tasks within 3 min. The TSST is a standardized procedure for inducing acute psychological stress and is considered the gold standard in stress research (137, 138). The core task, requiring participants to solve arithmetic problems quickly and accurately, effectively provoked stress by demanding complex calculations in a short time frame, leading to psychological tension and physiological responses, such as increased heart rate (139). This stress induction allowed for the assessment of whether exposure to NI could alleviate stress.

Given that epidemics act as chronic stressors, this study aimed to examine physiological and psychological responses under prolonged stress conditions (139, 140). By using controlled stress induction, the study explored the relationship between epidemics and stress, distinguishing between stress induced by experimental conditions and other factors like academic pressure.

After stress induction, participants completed the short-form Profile of Mood States (POMS) and State–Trait Anxiety Inventory (STAI) scales to assess their psychological state before exposure to NI. Trained staff then measured physiological indicators. Participants underwent a 15-min NI experience, followed by a second round of physiological and psychological assessments to evaluate changes.

Baseline data served as the control group, with no stress induction, while the experimental group examined changes after NI exposure under stress-induced conditions. This comparison enabled a valid assessment of the physiological and psychological effects of NI, helping to clarify the relationship between chronic stress from epidemics and stress induced by the experimental conditions.

### 2.5 Data analysis

#### 2.5.1 Environmental indicator data processing

Environmental data were processed using Microsoft Office Excel 2016 to calculate mean values and comfort indices for the six types of sample sites. The data were then analyzed with SPSS 27 to assess statistical significance. One-way ANOVA was used to determine differences between environmental conditions, with Duncan’s test applied for post-hoc comparisons when significant differences were found (*p* < 0.05). Graphs were created to visually represent the results and highlight differences between sample sites.

The Human Discomfort Index (DI) was evaluated based on four environmental parameters: thermal, air, light, and sound. The comfort index for the thermal environment was calculated using Equation 2 provided by the Beijing Meteorological Bureau.


(2) $$\begin{array}{c} DI=\left(1.818\;t+18.18\right)\;\left(0.88+0.002f\right)\\ +\left(t–32\right)/\left(45–t\right)–3.2v+18.2 \end{array}$$

This Equation 2 integrates temperature (t), relative humidity (f), and wind speed (v) to assess overall comfort in different environmental settings. The human thermal environment comfort level (see Table 2) was classified using a 9-level classification method (141). The concentration of Natural Assets Indicators (NAIs) was assessed following the Technical Specification for Observation of NAIs (LY/T2586 − 2016) (141), issued by the State Forestry Administration of the People’s Republic of China (PRC) in 2016 (see Table 3). The outdoor light environment (see Table 4) was evaluated based on the “Evaluation Method of Light Environment” (GB/T12454-2017), a national standard issued in 2017 (142, 143). The sound environment (see Table 5) was assessed according to the daytime noise limit values in the Sound Environment Quality Standard (GB3096-2008), issued by the Ministry of Ecology and Environment of the PRC in 2008 (144).

**Table 2:** Thermal environment comfort level criteria.

Human comfort level	Discomfort index	Feeling
4	≥86	Feeling extremely warm and extremely uncomfortable
3	80–85	Feeling very warm and uncomfortable
2	76–79	Feels warm and uncomfortable
1	71–75	Feels warm and comfortable
0	59–70	most comfortable
−1	41–58	Feels cooler and comfortable
−2	31–40	Feels cold and comfortable
−3	20–30	Feels cold and very uncomfortable
−4	≤ 20	Feels extremely cold and extremely uncomfortable

**Table 3:** NAIs level criteria.

Level	NAIs concentration (/cm^3^)	Level description
I	*n* ≥ 3,000	Extremely good
II	1,200 ≤ *n* < 3,000	Very good
III	500 ≤ *n* < 1,200	Good
IV	300 ≤ *n* < 500	Bad
V	100 ≤ *n* < 300	Very bad
VI	*n* < 100	Extremely bad

**Table 4:** Light level criteria.

Level	Level description	Illuminance (Lux)
I	Extremely comfortable	800–1,000
II	Very comfortable	600–800 or 1,000–1,200
III	Comfortable	450–600 or 1,200–1,350
IV	Uncomfortable	250–450 or 1,350–1,550
V	Very uncomfortable	< 250 or > 1,550

**Table 5:** Noise level criteria.

Level	Level description	Daytime noise limit (dB)
I	Comfortable	<50
II	Normal	50–55
III	Uncomfortable	55–65
IV	Very uncomfortable	>65

#### 2.5.2 Analysis of data on physical and psychological indicators of university students

SPSS software. These tools facilitated data synthesis and the calculation of changes in each index before and after the experience.

To assess the significance of differences in physiological indices among the six groups, one-way ANOVA was conducted in SPSS, followed by Duncan’s multiple comparison tests. This approach determined whether the observed changes were statistically significant, with a significance threshold set at *p* < 0.05. Standard errors were also calculated to assess the variability across sample groups.

The change in physiological and psychological indicators before and after the experience was calculated using the following Equation 3:


(3) N=m2–m1

N represents the change in each indicator, where m1 is the measurement before the experience, and m2 is the measurement after the experience.

A mixed research method was used to evaluate the effects of different NIs on physiological and psychological indicators. Changes in these indicators, both physiological and psychological, were quantified and analyzed using Pearson correlation coefficients. These coefficients assessed the relationships between changes in physiological indicators and changes in POMS and STAI scale scores. The analysis was conducted using SPSS, which provided correlation coefficients and *p*-values. To visualize the Pearson correlation analysis, correlation heatmaps were created using the *corrplot* package in R (version 4.1.1). This approach aimed to comprehensively assess the therapeutic benefits of different NIs and their combinations for human physiology and psychology.

## 3 Results

### 3.1 Influence of different NIs on human comfort

Compared to HI, NI offers more comfortable green spaces. Thermal comfort in NI ranges from 68.0 to 68.5, while HI averages 71.65. NI’s average temperature is 2–3°C cooler (17.1–17.6°C) due to BI and GI, whereas HI averages 20.8°C. NI also maintains higher humidity (69.7–74.9%) due to vegetation’s transpiration, while HI has lower humidity (59.5%) from increased evapotranspiration and reduced vegetation. Vegetation in NI acts as a windbreak, resulting in slower wind speeds (0.061–0.315 m/s), compared to the faster winds (0.663 m/s) in HI.

The air quality in NI benefits from higher concentrations of negative oxygen ions, which improve immunity and sleep. Forested areas within NI have the highest ion concentration (1,671 ions/cm^3^ on average), while other NI areas average 1,350 ions/cm^3^. In contrast, HI’s ion concentration is much lower (448 ions/cm^3^).

NI also provides better optical environments, with higher illumination levels, particularly in forests (1,010 lux on average), offering better protection from light pollution. In HI, hard landscaping results in higher artificial light levels (20,712 lux on average).

**Figure 3:**
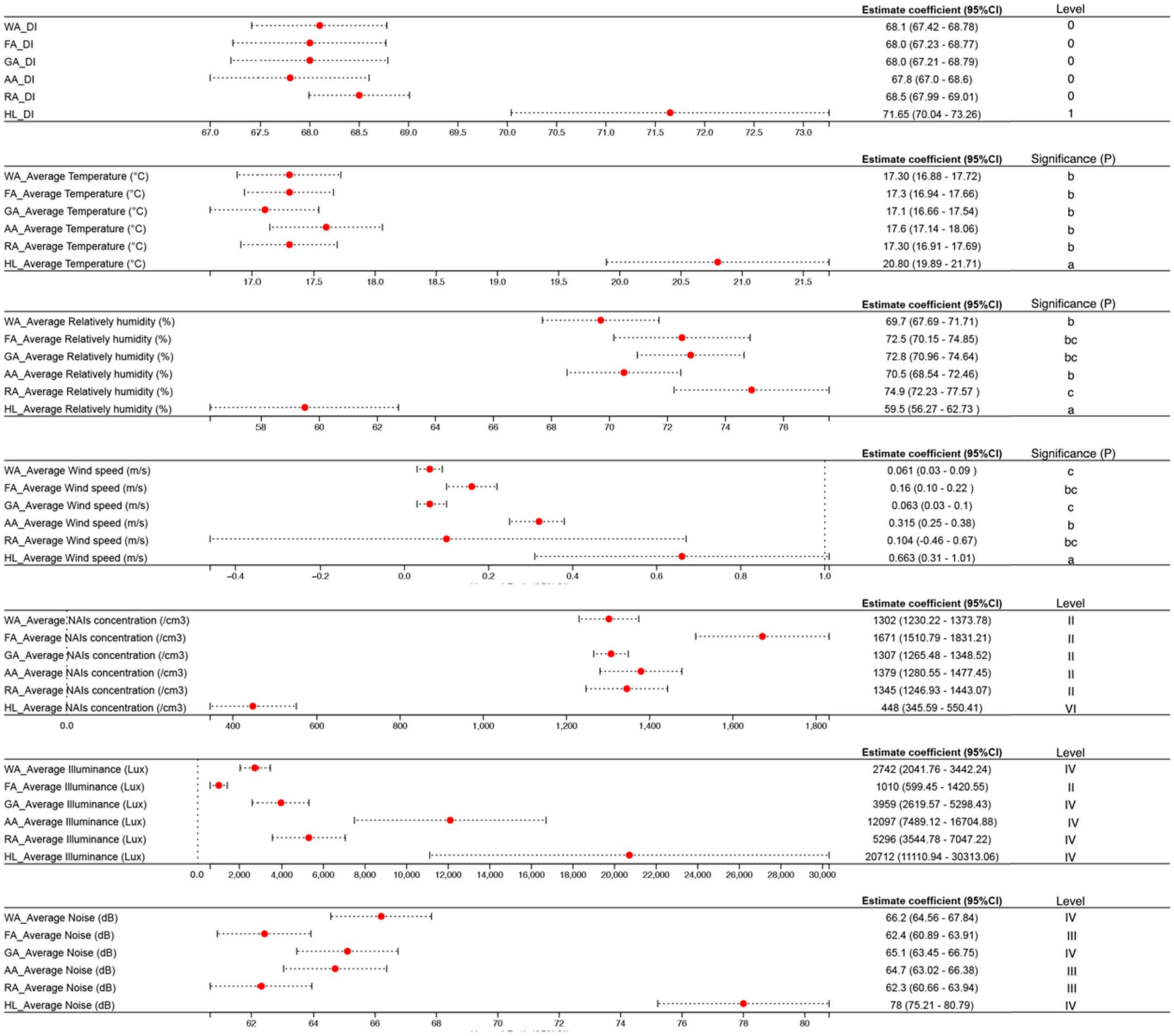
Environmental indicators for different natural infrastructures. Values in each column followed by the same letter are not significantly different at 0.05 level according to LSD (applies to Figures 2–4).

Acoustic environments significantly impact human comfort, with NI generally quieter (62.3–66.2 dB) compared to HI (78.0 dB). Overall, NI excels in providing better thermal comfort, air quality, light conditions, and quieter environments compared to HI, showcasing its potential to create more comfortable and healthier spaces. The pattern of change is as follows (Figure 3):

Thermal comfort: HL (71.65) > RA (68.5) > WA (68.1) > FA/GA (68.0) > AA (67.8).

Average Temperature (°C): HL (20.8) > AA (17.6) > WA/FA/RA (17.3) > GA (17.1).

Average Relatively humidity (%): RA (74.9) > GA (72.8) > FA (72.5) > AA (70.5) > WA (69.7) > HL (59.5).

Average Wind speed (m/s): HL (0.663) > AA (0.315) > FA (0.159) > RA (0.104) > GA (0.063) > WA (0.061).

Average NAIs concentration (/cm^3^): FA (1671) > AA (1379) > RA (1345) > GA (1307) > WA (1302) > HL (448).

Average Illuminance (Lux): HL (20712) > AA (12097) > RA (12097) > GA (3959) > WA (2742) > FA (1010).

### 3.2 Effects of different NIs on physiological and psychological indicators of university students

NI shows a more positive impact on physiological indicators compared to HI. This is particularly evident in the changes observed in systolic and diastolic blood pressure, heart rate, blood oxygen saturation, and pulse rate.

Systolic and Diastolic Blood Pressure Changes in these measures are significantly associated with the risk of developing cardiovascular diseases (97, 145). Higher blood pressure levels have been linked to increased cardiovascular risk, making these indicators critical for assessing overall cardiovascular health (146). Heart Rate serves as an important prognostic indicator both in the general population and among individuals with pre-existing cardiovascular conditions (147, 148). It is also a key measure in evaluating the effectiveness of exercise therapy for cardiovascular diseases (149). Blood Oxygen Saturation in the bloodstream are essential for maintaining normal metabolic functions and overall health. Low levels can impair bodily functions and indicate potential health issues. Pulse Rate helps in diagnosing various health conditions and reflects overall cardiovascular health (150). Changes in pulse rate can provide insights into the effectiveness of different types of natural infrastructure on physiological well-being.

Changes in physiological indices before and after the trial, along with standard errors for the six sample groups, were analyzed using SPSS 27.0 software. One-way ANOVA and Duncan’s multiple range test (MRT) were employed for this analysis. Each area experienced in NI resulted in varying decreases in systolic blood pressure, diastolic blood pressure, mean heart rate, and mean pulse rate among the subjects. In contrast, experiencing HI showed an increasing trend in these physiological indicators.

**Figure 4:**
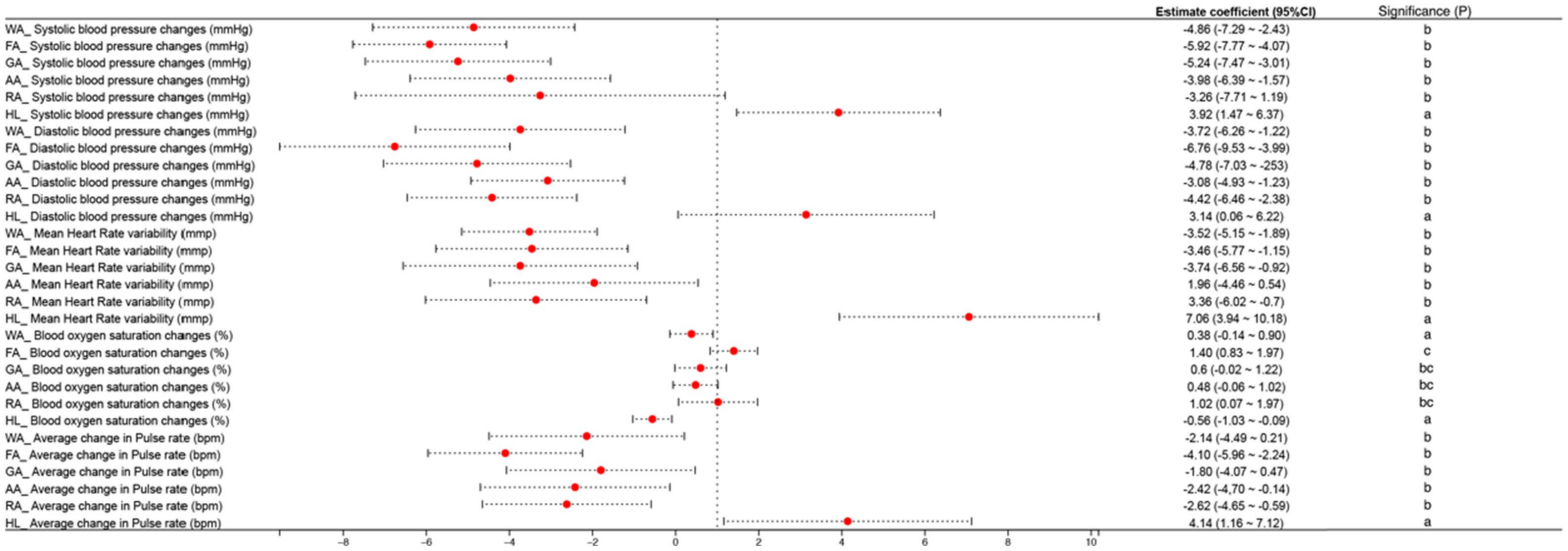
Effects of different natural infrastructures on changes in human physiological indicators.

After experiencing NI, subjects showed an upward trend in blood oxygen saturation, with the most significant effect observed as a 1.4% rise after exposure to the forest region. Conversely, experiencing HI resulted in a downward trend in blood oxygen saturation. NI demonstrates a distinct therapeutic function for the human body, where exposure to green spaces can foster non-pharmacological physical therapeutic effects that aid in recovery and overall health improvement. The observed pattern of change indicates that NI promotes physiological well-being more effectively compared to HI. The pattern of change is as follows (Figure 4):

Systolic blood pressure changes (mmHg): FA (− 5.92) > GA (− 5.24) > WA (− 4.86) > AA (− 3.98) > RA (− 3.26) > HL (+ 3.92).

Diastolic blood pressure changes (mmHg): FA (− 6.76) > GA (− 4.78) > RA (− 4.42) > WA (− 3.74) > AA (− 3.08) > HL (+ 3.14).

Mean Heart Rate variability: GA (− 3.74) > WA (− 3.52) > FA (− 3.46) > RA (− 3.36) > AA (− 1.96) > HL (+ 7.06).

Blood oxygen saturation changes (%): FA (+ 1.40) > RA (+ 1.02) > GA (+ 0.60) > AA (+0.48) > WA (+ 0.38) > HL (− 0.56).

Average change in Pulse rate (bpm): FA (− 4.10) > RA (− 2.62) > AA (− 2.42) > WA (− 2.14) > GA (− 1.80) > HL (+ 4.14).

The POMS form was utilized to assess psychological indicators following the experience. It comprises three dimensions: euphoria, relaxation, and agitation, scored on a 7-point Likert scale where higher scores indicate a more pronounced state. The TMD score reflects the intensity of negative emotions and mood instability.

The STAI includes two distinct self-assessment questionnaires. One evaluates individuals’ “right now” feelings (“state anxiety”), while the other assesses their enduring predisposition to anxiety (“trait anxiety”).

**Figure 5:**
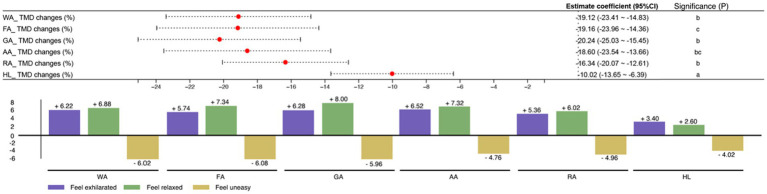
Impact of natural infrastructures on psychological indicators assessed by POMS.

NI was more effective than HI in enhancing the psychological state of the human body. While changes in TMD generally decreased in all subjects following both NI and HI experiences, NI proved relatively more effective (refer to Figure 5). Specifically, the GA experience showed the greatest effectiveness in improving TMD status (−20.24), followed by WA (−19.12) and FA (−19.16). In contrast, the experience of Hard Landscaping exhibited the lowest improvement among the six areas (−10.02).

The experiences of NI and HI yielded positive outcomes across all three assessment dimensions, indicating beneficial effects. Specifically, there was an increase in subjects feeling exhilarated after the NI experience, with Agricultural Areas showing the most significant improvement (+6.52), and Hard Landscaping demonstrating a slight enhancement (+3.40). Regarding feeling relaxed, NI had the most pronounced effect in Grass Areas (+8.00), whereas Residential Areas showed the highest improvement in HI (+6.02). Although HI also showed an upward trend in feeling relaxed (+2.60), it was markedly less effective compared to NI.

University students feeling uneasy experienced effective improvements in Forest Areas after experiencing NI (−6.08), whereas HI also improved this dimension (−4.02), albeit less effectively than NI. Specifically, Agricultural Areas in NI showed a better effect (−4.76) compared to HI (−4.02).

**Figure 6:**
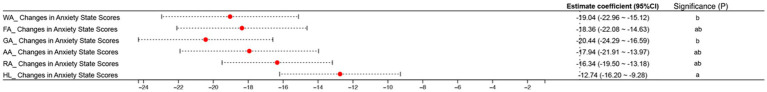
Impact of natural infrastructures on psychological indicators assessed by STAI.

Although both NI and HI effectively alleviate subjects’ anxiety states before and after the experience, NI consistently outperformed HI in both STAI assessments (refer to Figure 6). The most effective anxiety relief in NI was observed in Grass Areas, where it significantly reduced anxiety (−20.44). In contrast, HI demonstrated an anxiety-relieving effect (−12.74), slightly lower than the effect observed in RA in NI (−16.34). Overall, the NI experience proved more effective than HI in improving the psychological state and reducing anxiety among the subjects. The specific patterns of change were as follows (Figure 6):

TMD changes (%): GA (−20.24) > FA (−19.16) > WA (−19.12) > AA (−18.60) > RA (−16.34) > HL (−10.02).

Feel exhilarated: AA (+6.52) > GA (+6.28) > WA (+6.22) > FA (+5.74) > RA (+5.36) > HL (+3.40).

Feeling relaxed: GA (+8.00) > FA (+7.34) > AA (+7.32) > WA (+6.88) > RA (+6.02) > HL (+2.60).

Feel uneasy: FA (−6.08) > WA (−6.02) > GA (−5.96) > RA (−4.96) > AA (−4.76) > HL (−4.02).

Changes in Anxiety State Scores: GA (−4.02) > WA (−19.04) > FA (−18.36) > AA (−17.94) > RA (−16.34) > HL (−12.74).

### 3.3 Correlation of different NIs on human physiological and psychological indicators

**Figure 7:**
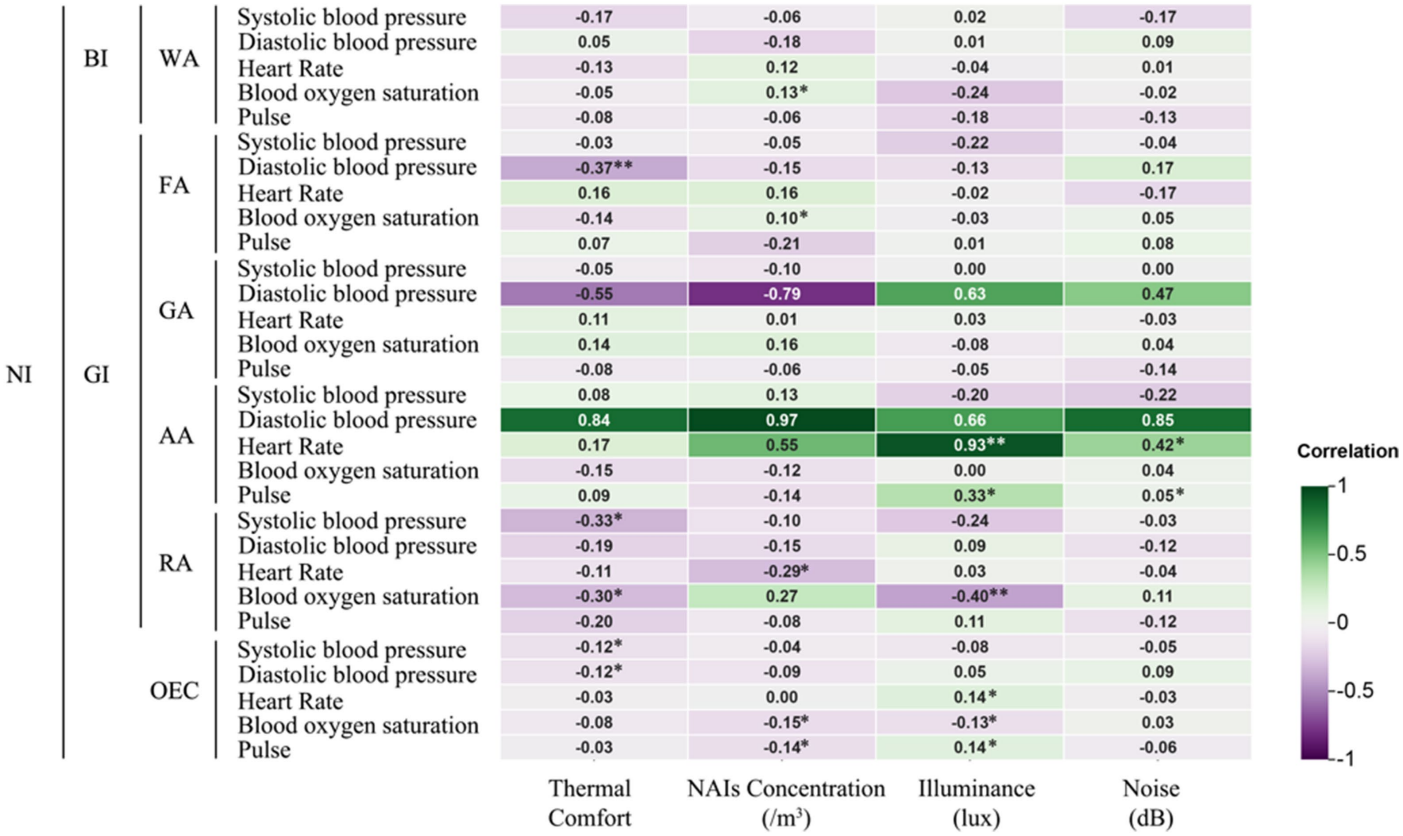
Correlation of different natural infrastructures on human physiological indicators. *Indicates a significant correlation at the 0.05 level; **indicates a significant correlation at the 0.01 level (applies to Figures 8–10).

**Figure 8:**
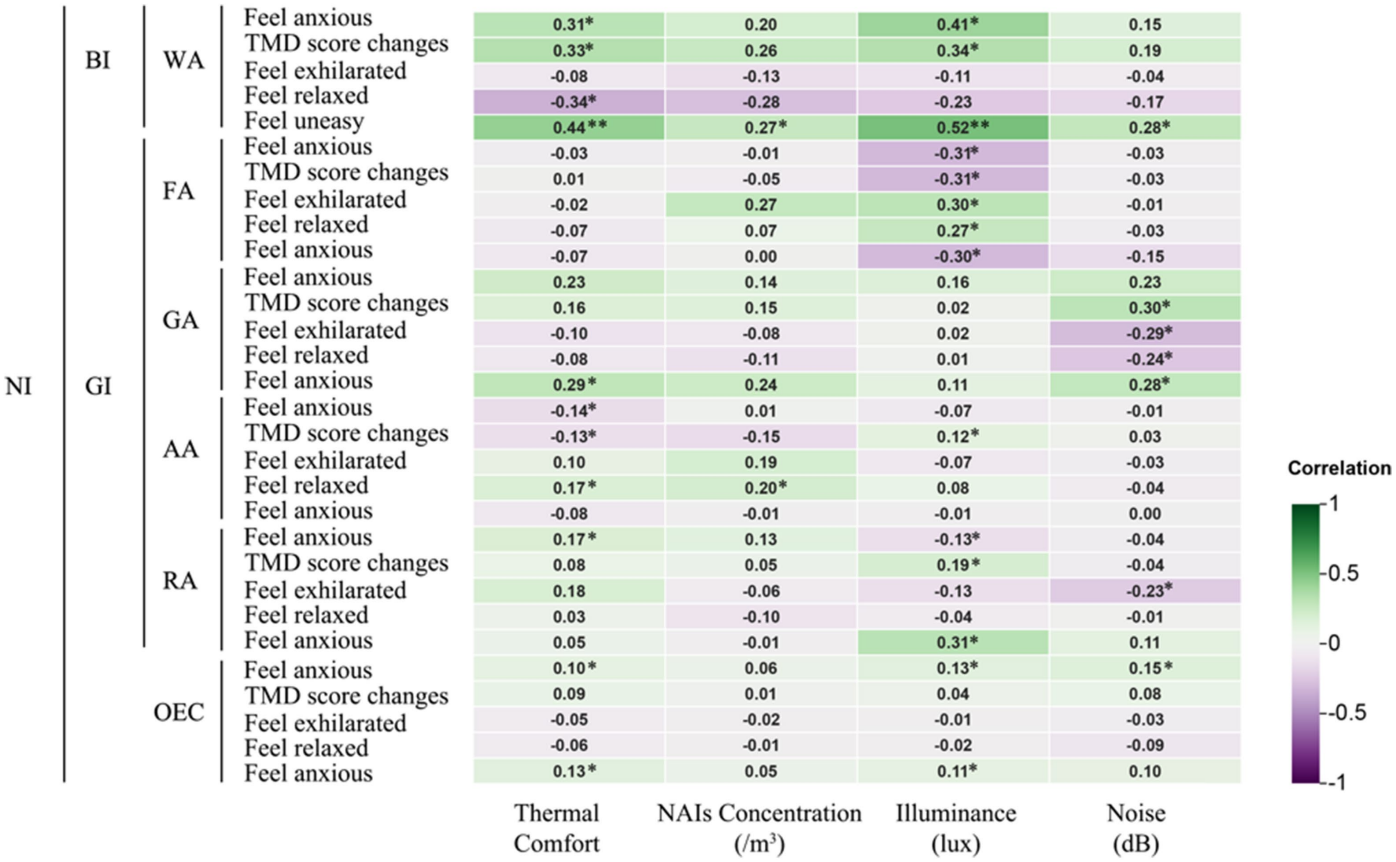
Correlation of different natural infrastructures on human psychological indicators.

The study found that environmental factors such as thermal comfort, NAIs, illuminance, and noise in different NIs significantly affect human physiological and psychological indices. Figure 7 shows a positive correlation (*p* < 0.05) between blood oxygen saturation and NAIs concentration following the WA experience. Figure 8 reveals that feelings of “Anxiety” (*p* < 0.05) and “Unease” (*p* < 0.01), as well as changes in TMD values (*p* < 0.05), were positively correlated with thermal comfort after the WA experience. Conversely, “Relaxation” (*p* < 0.05) had a negative correlation with thermal comfort. Light levels were positively correlated with “Anxiety” (*p* < 0.05) and “Unease” (*p* < 0.01) and influenced TMD changes (*p* < 0.05). Noise levels were positively correlated with “Unease” (*p* < 0.05). The WA environment, with abundant vegetation, lower temperatures, high NAIs, favorable thermal comfort, adequate illuminance, and lower noise levels, positively affected blood oxygen levels and reduced negative perceptions.

After experiencing the FA, systolic blood pressure was significantly negatively correlated with thermal comfort (*p* < 0.01), indicating that higher thermal comfort was associated with lower blood pressure, enhancing student comfort. NAIs concentration was positively correlated with both blood oxygen saturation and feelings of exhilaration (*p* < 0.05). Light levels were positively correlated with anxiety, unease, and TMD values (*p* < 0.05), but also with exhilaration and relaxation (*p* < 0.05). Dense vegetation and high NAIs in the FA increased blood oxygen saturation and exhilaration. However, the sense of closure and lower light levels in the FA promoted relaxation but also heightened anxiety, unease, and TMD values.

In the GA, no significant correlations were found between physiological indicators and environmental factors. However, thermal comfort in the GA was positively correlated with feelings of unease (*p* < 0.05), while noise levels negatively correlated with exhilaration (*p* < 0.05), and TMD values were positively correlated with unease (*p* < 0.05). The less dense vegetation and greater openness of the GA reduced its ability to enhance environmental conditions, with increased noise and thermal comfort fluctuations contributing to negative perceptions and TMD changes.

After the AA experience, heart rate was significantly positively correlated with both light levels (*p* < 0.01) and noise levels (*p* < 0.05), while pulse rate was also positively correlated with both (*p* < 0.05). Thermal comfort was negatively correlated with feelings of anxiety and changes in TMD values (*p* < 0.05), but positively correlated with relaxation (*p* < 0.05). NAIs concentration in the AA was significantly correlated with relaxation (*p* < 0.05).

The AA, which mainly consisted of farmland with few tall buildings or trees, showed that higher noise levels were linked to increased heart rate and pulse rate. The lack of vegetation and high light levels also contributed to faster pulse rates. Thermal comfort had a significant impact on subjects’ perceptions: deviations from optimal comfort increased anxiety and TMD changes, while closer alignment with the ideal thermal range promoted relaxation.

In the RA, thermal comfort was negatively correlated with both systolic blood pressure and blood oxygen saturation (*p* < 0.05). NAIs concentration showed a significant negative correlation with heart rate (*p* < 0.05) and a stronger negative correlation with blood oxygen saturation (*p* < 0.01). Light levels were positively correlated with blood oxygen saturation (*p* < 0.01) but negatively correlated with feelings of anxiety (*p* < 0.05). Light levels were also positively correlated with changes in TMD values and feelings of unease (*p* < 0.05), while noise levels were negatively correlated with feelings of exhilaration (*p* < 0.05).

The RA’s limited vegetation failed to improve the microclimate, negatively impacting blood pressure and blood oxygen saturation. The prevalence of artificial lighting and increased noise levels worsened physiological and psychological indicators, exacerbating negative perceptions and lowering blood oxygen saturation.

**Figure 9:**
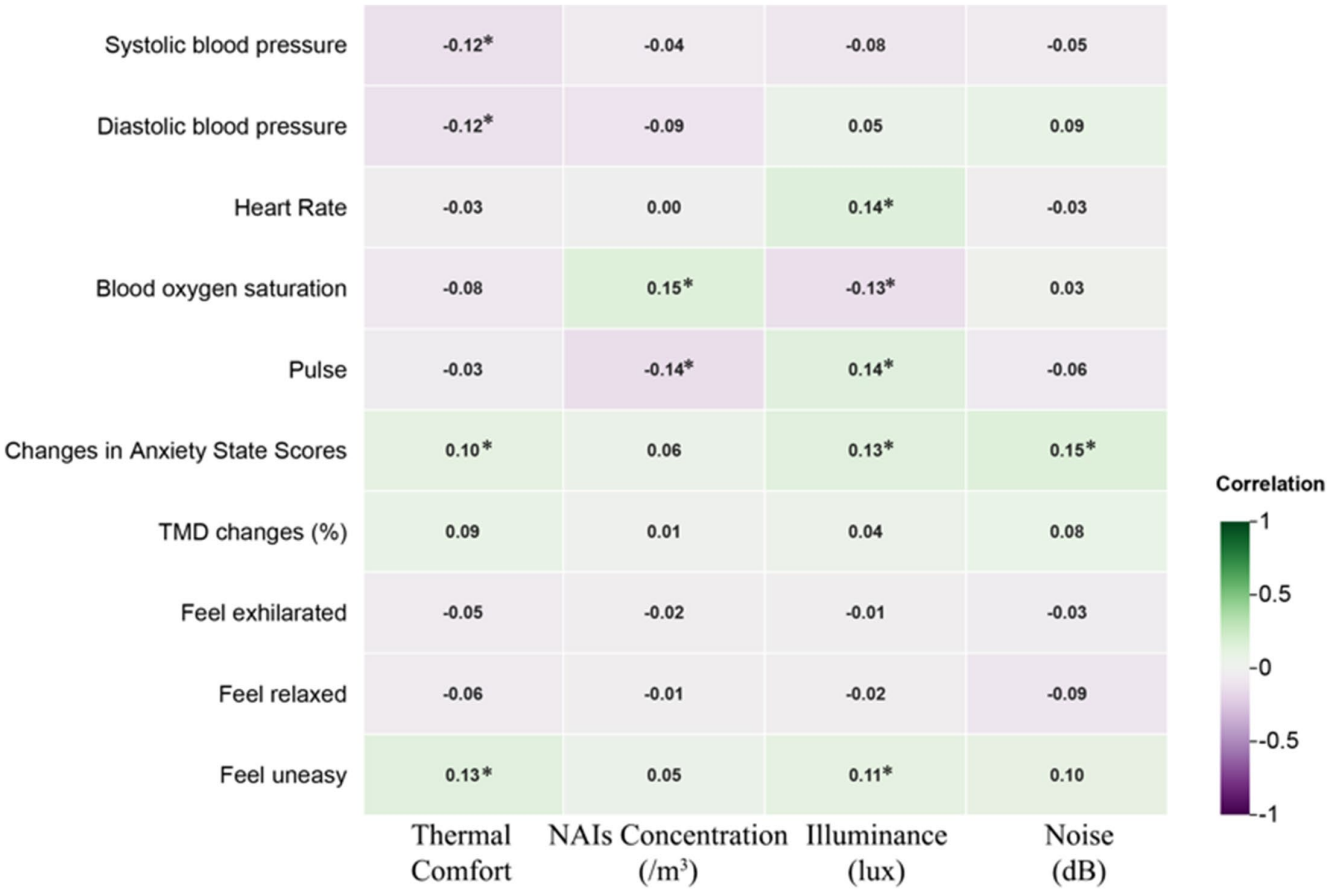
Effects of the natural infrastructure on the correlation of physiological and psychological indicators.

Changes in both diastolic and systolic blood pressure were significantly negatively correlated with thermal comfort, particularly in the FA (*p* < 0.05) and AA (*p* < 0.01) experiences (Figure 9). Alterations in the thermal environment not only affected blood pressure but also contributed to psychological issues. In the WA, RA, and AA, where thermal comfort was closest to the ideal range, reductions in negative emotions like anxiety were observed (−19.04, −16.34, and −17.9, respectively). Both WA and GA experiences helped alleviate feelings of unease (−6.02 and −5.96, respectively). TMD values also decreased significantly after WA and AA (−19.12 and −18.60), while feelings of relaxation increased notably (+6.88 and +7.32). These psychological improvements were significantly correlated with thermal comfort, highlighting its direct impact on both blood pressure and psychological states like anxiety and unease.

**Figure 10:**
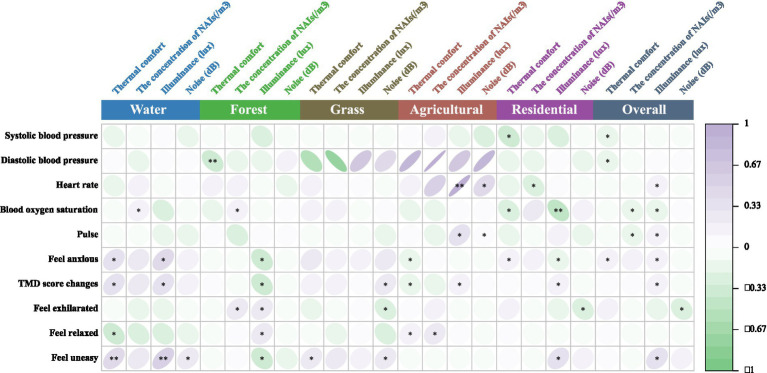
Pearson correlation analysis of physiological and psychological in different environments. Pearson correlation coefficients range from −1 to 1. A coefficient between 0 and 0.33 indicates a weak correlation, between 0.33 and 0.67 indicates a moderate correlation, and between 0.67 and 1 indicates a strong correlation. A single asterisk (*) denotes a significance level of *p* < 0.05, while two asterisks (**) denote a significance level of *p* < 0.01.

Figure 10 presents the Pearson correlation matrix, illustrating the effects of various NI environments on physiological and psychological changes in university students. The heatmap uses color coding—green for negative correlations and purple for positive correlations—where the intensity of the color represents the strength of the correlation. This visual format allows for quick identification of the relationships and their magnitudes.

In the WA, thermal comfort and illumination showed a moderate positive correlation with anxiety and unease, suggesting that higher levels may exacerbate these emotions. Conversely, they were moderately negatively correlated with relaxation, indicating reduced relaxation under such conditions. In the FA, increased thermal comfort was moderately negatively correlated with diastolic blood pressure, while higher illumination levels showed a moderate negative correlation with anxiety, unease, and TMD values, suggesting potential alleviation of these symptoms.

In the GA, thermal comfort and NAIs had a strong negative correlation with diastolic blood pressure, indicating that higher thermal comfort and NAIs concentration lowered blood pressure. However, illuminance was strongly positively correlated with diastolic blood pressure, suggesting an opposite effect. In the AA, thermal comfort, NAIs, illuminance, and noise were strongly positively correlated with heart and pulse rates, with illuminance moderately correlated with pulse rate and noise moderately correlated with heart rate, indicating increased cardiovascular activity under these conditions.

In the RA, higher thermal comfort was moderately negatively correlated with systolic blood pressure and blood oxygen saturation, while illuminance was strongly negatively correlated with blood oxygen saturation, highlighting its significant impact on physiological parameters. Additionally, illuminance showed a moderate positive correlation with unease across environments, while noise was moderately negatively correlated with exhilaration, indicating a reduction in pleasure with higher noise levels.

These findings underscore the nuanced impacts of environmental factors like thermal comfort, illumination, NAIs, and noise on university students’ physiological and psychological states, offering actionable insights to improve environmental conditions for enhanced health and well-being.

**Figure 11:**
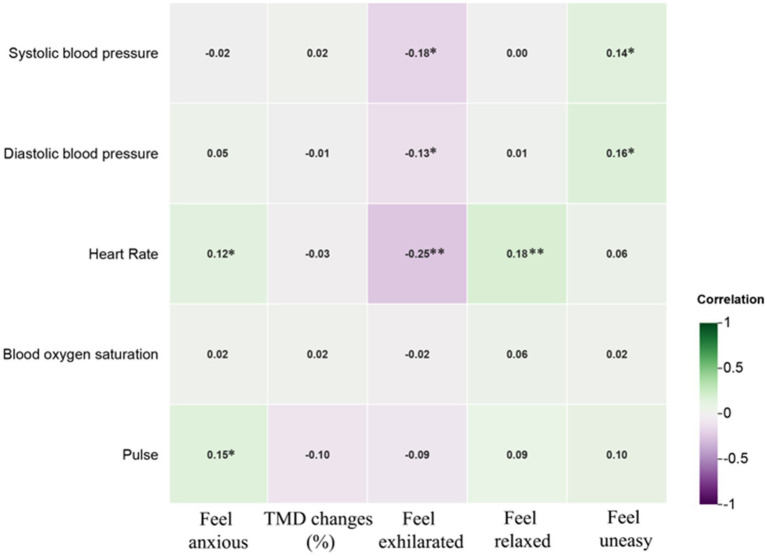
Correlation of physiological and psychological indicators.

Research indicates that paying attention to negative emotions can positively impact psychological health, with such attention potentially alleviating these emotions (151, 152). Conversely, ignoring negative emotions may lead to their intensification (153, 154). However, some studies dispute the connection between negative emotions and psychological health (155). In addition, heart Rate Variability (HRV) was found to be significantly positively correlated with feelings of anxiety (*p* < 0.05) and unease (*p* < 0.01) among university students (refer to Figure 11). Experiences in HL led to an increase in HRV (+7.06), while NI experiences generally resulted in a decrease in HRV. In AA, HRV showed a significant positive correlation with light (*p* < 0.01) and noise levels (*p* < 0.05), whereas in RA, the concentration of NAIs was significantly negatively correlated with HRV (*p* < 0.05). Previous research has associated higher HRV with improved psychological and physical health (156, 157). Thus, NI experiences may influence HRV, thereby enhancing psychological health, with higher concentrations of NAIs potentially providing greater therapeutic benefits.

Extensive research on NAIs is crucial for evaluating the effectiveness of green spaces in enhancing human physical and psychological health (158, 159). Blood oxygen saturation was significantly positively correlated with NAIs (*p* < 0.05). NAIs in WA and FA were significantly associated with increased blood oxygen saturation levels among university students (*p* < 0.05). There were notable increases in oxygen saturation levels after students experienced WA and FA, with changes of +0.38 and +1.40, respectively. FA demonstrated the most substantial positive effect of NAIs on students’ oxygen saturation. Additionally, there was a significant increase in the feeling of exhilaration among students after experiencing FA (+5.74), which was also positively correlated (*p* < 0.05). Environments with high concentrations of NAIs tend to enhance blood oxygen levels, which positively impacts fatigue relief, sleep quality, and respiratory function, while reducing negative emotional perceptions and lowering the risk of anxiety and depression.

Noise is a significant environmental factor that impacts human health and well-being, with efforts often focused on mitigating its negative effects (160). Research has shown that increased noise levels heighten feelings of anxiety. In particular, the noise levels in WA and GA were associated with increased negative perceptions among university students, with correlations showing a significant positive relationship with feelings of unease (*p* < 0.05). Additionally, noise levels in GA were significantly correlated with changes in TMD values, indicating a marked negative impact on mood (*p* < 0.05). Conversely, higher noise levels in RA were negatively correlated with feelings of exhilaration among students (*p* < 0.05).

## 4 Discussion

While most individuals infected with COVID-19 recover fully, evidence indicates that approximately 10–20% experience a range of medium- to long-term effects following the initial illness (160). These symptoms, which can persist from the initial onset or appear after recovery, may fluctuate over time, including symptoms such as fatigue, shortness of breath, and cognitive dysfunction (e.g., confusion, forgetfulness, or lack of concentration). These persistent issues can significantly impact individuals’ daily lives. In response, the WHO introduced the ICD-10 code (U09) and the ICD-11 code (RA02) in September 2020 to address and monitor the long-term effects of COVID-19 (161). Despite this, there remains limited information on the long-term outcomes of COVID-19. To address this gap, the WHO’s Clinical Management and Operations Unit has launched a series of online lectures aimed at creating a global network of clinicians to better identify, diagnose, and treat long-term COVID-19 symptoms (161, 162). This initiative provides a valuable opportunity for individuals with persistent symptoms, particularly those lacking financial resources, to access much-needed rehabilitation and support until a medical cure is found.

Common symptoms of post-COVID-19 conditions include fatigue, shortness of breath, memory and concentration issues, sleep disturbances, and symptoms of depression or anxiety (WHO, 2023). While anyone can develop these sequelae, studies indicate that approximately 10–20% of patients may experience long-term effects, and the duration of these symptoms is difficult to predict (WHO, 2023). The findings suggest that integrating natural infrastructures into daily life might help alleviate some of these effects, potentially benefiting university students.

Overall, this study highlights the substantial practical benefits of NI for the physiological and psychological well-being of university students, especially in the post-pandemic era. It demonstrates that NI offers valuable non-pharmacological options for addressing health issues and emotional challenges arising from the pandemic. Both male and female students of various ages acknowledged the therapeutic potential of NI experiences. The findings reveal significant variations in the effectiveness of different NIs in mitigating physical health problems and negative emotions related to the pandemic’s aftermath.

Common post-COVID-19 complications can lead to a range of issues including hypertension, increased heart rate, low oxygen saturation, rapid pulse rate, and psychological problems such as anxiety and depression. Hypertension is a major preventable risk factor for death; however, only about one-third of patients manage to control their blood pressure effectively (61). Elevated blood pressure is a leading global risk factor for mortality (163). Despite advances in treatment, achieving effective blood pressure control remains a significant global challenge (164–168). The blood pressure health of university students, in particularly, is often neglected (169).

Interestingly, the warmer environments in FA and RA proved most effective in reducing both diastolic and systolic blood pressure among university students. These findings align with previous research that highlights the potential of green spaces to mitigate hypertension risk (158, 170–172). Conversely, younger individuals, whose blood pressure fluctuates more in FA, may experience greater benefits from green spaces compared to other studies (173). Additionally, WA, AA, and RA environments were effective in improving negative moods among university students, consistent with findings from other studies (174–176).

Additionally, university students’ perceptions of negative emotions are influenced by HRV. Various infrastructures, such as HL, contribute to an increase in HRV among university students, while the NI environment has a beneficial effect on HRV. This supports the findings of Quirin et al. (177), who identified a plausible link between HRV and psychological health. Previous research has shown that higher HRV is associated with better psychological and physical health outcomes (156, 157). In AA, light and noise levels positively influence HRV, whereas in RA, higher concentrations of NAIs have been linked to negative changes in HRV.

Consistent with previous research, this study confirms the positive relationship between NAIs and blood oxygen saturation (178). Significant increases in blood oxygen saturation were observed in college students after exposure to WA and FA environments. Although this study did not establish a direct link between NAIs and hypertension, as noted by Chen et al. (158), it is widely accepted that maintaining good air quality and a healthy living environment is crucial for improving blood oxygen saturation.

Furthermore, NAIs contribute to restoring vitality among college students. Environments with high concentrations of negative oxygen ions tend to increase blood oxygen levels, which positively affects fatigue reduction, improves sleep quality, alleviates dyspnoea, diminishes negative emotional perceptions, and lowers the risk of anxiety and depression.

Few studies have directly examined the impact of green space light levels on human health, though related research has addressed this issue indirectly. Lai et al. (179) found that tree shade reduces average radiant temperature by diminishing shortwave solar radiation (179). Similarly, Elsadek et al. (180) demonstrated that optimizing tree layout to provide better shade can alleviate discomfort. Javadi (181) assessed how appropriate shade coverage can enhance the positive health impacts for urban residents.

This study contributes to this body of knowledge by exploring how different light levels in NI correlate with emotional perceptions. It confirms the impact of light levels on psychological health, further validating the role of environmental light in influencing psychological well-being.

The study highlights the significant impact of noise on health, both physical and psychological. Prior research, such as that by Stansfeld et al. (160), has investigated various health responses to noise, particularly among vulnerable groups. This study builds on these findings by examining the specific health effects of different types and intensities of noise, providing insights into potential mitigation strategies and policy recommendations.

The results confirm that noise significantly influences anxiety levels, with louder noise intensifying feelings of anxiety. Additionally, Bloemsma et al. (182) underscore the detrimental effects of traffic noise on adolescent psychological health, further supporting the findings of this study regarding the adverse impacts of noise on psychological well-being.

These studies underscore the substantial therapeutic benefits of NI for university students, particularly in the post-pandemic era. NI proves to be an invaluable resource for students who are dealing with persistent symptoms and those with limited financial resources. The positive impact of NI on the physical and psychological well-being of university students, aged 18–25, is evident.

During the pandemic, many students experienced significant stress, with over half (53.6%) reporting a strong sense of pressure before engaging with NI. However, after experiencing NI, 65.6% of students felt relief from stress, and 72.8% recognized the importance of therapeutic outdoor experiences. These findings align with research on the impact of the pandemic on young people’s daily lives and highlight the therapeutic value of green spaces, as noted by Goodenough et al. (183), Vos et al. (184), and Pipitone and Jović (185). The study’s results reinforce the idea that spending time in various natural environments can significantly alleviate stress, as reflected in the students’ enthusiasm for future outdoor activities (183–185). This is particularly evident from the questionnaire response indicating that university students express “a desire to engage in outdoor activities and explore diverse natural environments in the future.”

Even prior to the outbreak, numerous studies had explored the therapeutic effects of exposure to green spaces (83, 186, 187). This ongoing scholarly interest highlights the recognized physiological and psychological benefits of green spaces and underscores the increased acknowledgment of the therapeutic value of various NIs within these environments, especially in the wake of the epidemic. This highlights the critical importance of accessibility to urban green spaces. Enhancing green connectivity across different urban areas ensures that every community has access to natural spaces. Future urban planning should prioritize the strategic integration of natural infrastructure within cities to meet the diverse needs of various populations and encourage individuals to spend more time outdoors. Equally important is improving the environmental quality of urban green spaces to maximize their therapeutic effects, collectively contributing to the recovery from health challenges in the post-pandemic era.

## 5 Limitation

However, the study did not separately account for the therapeutic effects on males and females. It is recognized that the benefits of different NIs may vary between genders. For instance, Wang et al. (188) found that women may experience greater benefits from street view greenspace (SVG) compared to men, particularly in relation to hypertension (188). Supporting this, studies conducted in China (108) and Austria (189) also indicate that women tend to gain more from exposure to green spaces. Conversely, research by Jiang et al. (190) suggests that men might benefit more from outdoor green spaces. Additionally, a comparative study in Belgium and Spain by Bauwelinck et al. (191) found no significant gender differences in the use of green spaces.

More research indicates that women generally experience more significant benefits from green spaces compared to men (121, 189, 192), potentially due to enhanced health benefits (193). Studies by Sang et al. (193) and Shen et al. (194) suggest that women often have stronger visual perceptions and more positive responses to green spaces, including heightened olfactory sensitivity, which may enhance the therapeutic effects of these environments. Additionally, women are typically more likely to engage in positive activities such as exercising in green spaces, whereas men may prefer to stay within residential areas (193, 195, 196). These factors could contribute to the observed differences in how green spaces impact male and female health.

## 6 Conclusion

This study highlights the critical role of diverse natural environments in supporting the mental and physical health of university students during the pandemic. The research shows that for students aged 18 to 25, increasing outdoor activities and exposure to different NIs can significantly improve their well-being. Most participants reported enhancements in their physical, psychological, and emotional health through interactions with nature. However, a subset of students continued to experience lingering effects from the pandemic, reporting varied therapeutic outcomes. They noted that not all natural environments were equally effective, and issues such as fatigue, insomnia, and anxiety persisted.

The findings reveal not only the immediate benefits of engaging with NIs during the pandemic but also suggest that these interactions may have lasting impacts on students’ well-being post-pandemic. Although the data was collected during the pandemic, the observed effects may hold long-term significance, as the health and well-being challenges posed by the pandemic may not dissipate quickly but could continue or evolve over time. The study underscores the immediate and potential long-lasting benefits of natural infrastructures for the health and well-being of Chinese university students.

Therefore, this study advocates for the integration of diverse natural infrastructures into future urban planning to meet the varied needs and preferences of different populations. By providing a range of natural environments, this research supports the recovery of specific groups, addresses the health challenges posed by the pandemic, promotes outdoor activities, and emphasizes the crucial role of green spaces in enhancing the health and well-being of young university students.

## Data Availability

The original contributions presented in the study are included in the article/supplementary material, further inquiries can be directed to the corresponding author.
